# Impact of dry eye disease treatment on patient quality of life

**DOI:** 10.3389/fmed.2024.1305579

**Published:** 2024-02-28

**Authors:** Cheng-Wei Lin, Meng-Yin Lin, Jin-Wei Huang, Tsung-Jen Wang, I-Chan Lin

**Affiliations:** ^1^School of Medicine, College of Medicine, Taipei Medical University, Taipei, Taiwan; ^2^Department of Ophthalmology, Taipei Medical University, Shuang Ho Hospital, New Taipei City, Taiwan; ^3^Department of Ophthalmology, School of Medicine, College of Medicine, Taipei Medical University, Taipei, Taiwan; ^4^Department of Ophthalmology, Hualien Tzu Chi Hospital, Hualien, Taiwan; ^5^Department of Ophthalmology, Taipei Medical University Hospital, Taipei, Taiwan; ^6^Department of Ophthalmology, Wan Fang Hospital, Taipei Medical University, Taipei City, Taiwan

**Keywords:** dry eye diseases, quality of life, patient-reported outcomes, treatment, impact

## Abstract

Dry eye disease (DED) is a common multifactorial disease affecting a substantial proportion of the population worldwide. Objective tests and subjective symptoms evaluation are necessary to assess DED. Although various treatments have been introduced, accurately evaluating the efficacy of those treatments is difficult because of the disparity between diagnostic tests and patient-reported symptoms. We reviewed the questionnaires used to evaluate DED and the improvements of quality of life with various treatments. In addition, we highlighted the importance of patient-reported outcomes (PRO) assessments for evaluating the effect of DED treatments. Given that the assessment of DED treatment effectiveness substantially relies on individual ocular experiences, acquiring qualitative PRO data is essential for comprehensive evaluation and optimal treatment management. Clinicians should not only focus on improving objective symptoms but also prioritize the well-being of patients in clinical management.

## Introduction

Dry eye disease (DED) is a common ocular surface disease that affects a substantial proportion of the population worldwide. The prevalence of DED varies across different regions, ranging from 4.6% in North America to 47.9% in Africa (1). In Asian countries, approximately 20.1% of individuals develop DED (2). Moreover, in some industrialized Asian countries, such as Taiwan (3), Korea (4), and Japan (5), over a quarter of the population is affected.

DED is a multifactorial disease characterized by an imbalance between insufficient aqueous production (6) and excessive tear evaporation (7). Decreased tear production by the lacrimal gland results in less eye surface lubrication, and decreased oil secretion by the meibomian gland leads to excessive tear evaporation (8). The decreased wettability type of DED is characterized by a short tear film break-up time (TBUT), normal tear production, and minimal or no staining. This type results from the deficiency or abnormality of membrane-associated mucin, causing impaired corneal surface wettability (9). The most common risk factors with the strongest contribution for DED include female sex, contact lens usage, prolonged computer use, thyroid abnormalities, hypertension, antidepressant use, and antihistamine use (2). Other risk factors include Asian ethnicity (10), hormonal dysfunction and replacement therapy (11), Sjögren’s syndrome (12, 13), lifestyle factors (14), aging (2, 15, 16), medication usage, and cataract surgery (17, 18). These factors contribute to tear film instability, hyperosmolarity, ocular surface inflammation, and subsequent ocular discomfort (19).

Previously, DED was mainly attributed to aqueous insufficiency and ocular surface inflammation. Recent research has indicated meibomian gland dysfunction (MGD) as the leading cause of DED, particularly evaporative DED, and aqueous-deficient dry eye may be caused by MGD (5, 7). Thus, new diagnostic assessments and therapeutic interventions have been developed to address MGD (5, 20, 21) and restore the homeostasis of the tear film.

Objective tests and subjective symptom examination are mandatory for the accurate diagnosis of DED. However, disparities between diagnostic tests and patient-reported symptoms have been reported because of varied etiologies and clinical presentations (22–24). By evaluating patients’ symptoms and quality of life (QoL), the effect of the disease on individuals can be determined. Currently, no single test is available that can precisely predict and evaluate an individual’s response to treatment. Therefore, a standardized classification system that combines objective measurements with subjective symptom assessment and functional lifestyle evaluation through the use of well-designed questionnaires has been recommended to guide treatment strategies (19, 24, 25).

Various questionnaires have been developed to examine patient-reported outcomes (PROs) and the subjective symptoms of DED. Herein, we review the efficacy of conventional and advanced therapies as well as procedures (punctual occlusion, thermal pulsation, and intense pulsed light) in alleviating clinical signs and patient-reported symptoms. In addition, we evaluated questionnaires used to examine subjective ocular symptoms and QoL.

In this review, we evaluated the literature on the effect of current DED treatments on subjective outcomes. Given that subjective symptoms do not consistently correlate with objective clinical advancements, we focused on investigating the effects of treatments on the basis of patients’ self-reported improvements, encompassing self-reported symptoms, and satisfaction levels and by using validated questionnaires. By examining patients’ subjective responses to various treatment modalities, we intended to provide practitioners with valuable references for making informed treatment decisions. In our data search for clinical-trial-based articles, we initially employed specific commercial products or ingredients as primary search terms. Subsequently, we complemented our search by including the terms “dry eye” and “subjective” to refine and identify targeted search results. We comprehensively searched reputable databases, such as PubMed, Medline, and Web of Science, for relevant published studies related to DED treatments and their subjective impact. All articles meeting our search criteria that were published between 2000 and November 2022 (*n* = 9,050) were meticulously analyzed to identify clinical-trial-based publications focusing on assessment of QoL and subjective outcomes in human (*in vivo*) studies. With careful consideration, relevant articles investigating DED treatments and subjective assessments were selected, and their full contents were thoroughly evaluated (*n* = 255). The subsequent sections elucidate specific treatments for DED, including a detailed evaluation of their effects on QoL and patient satisfaction. We included not only original research papers but also other types of papers, such as trials and reviews, examining treatments for DED and questionnaires used to evaluate the QoL of patients with DED. We review studies on questionnaires and assessment tools for DED, and discuss the treatment options for DED. In addition, we discuss the advantages and disadvantages of possible treatment options for DED through comparative analysis.

## Review on DED treatments and subjective assessments

### Questionnaires and assessment tools for DED and ocular symptoms

PROs are highly valuable references because they directly capture the patient’s perspective without any interpretation from clinicians or third parties (26). Quantitative measurements alone may not always provide a definitive diagnosis of DED (27). Therefore, well-designed PRO instruments can provide complementary information and a more comprehensive understanding of patients’ condition (28). In addition to investigating the effect of DED or the effectiveness of its treatment, evaluating treatment satisfaction on the basis of direct patient feedback is essential. This evaluation can determine the effectiveness of treatment in alleviating symptoms as well as its convenience and accessibility.

Our review revealed various questionnaires and assessment tools that have been employed to differentiate patients with DED from those with normal ocular health and to capture subjective treatment outcomes. We categorized these questionnaires into two groups on the basis of their intended purpose: subjective ocular symptom measurement and QoL assessment. Because both groups of questionnaires rely on the subjective responses of individual patients, we compiled a table to differentiate the characteristics and purposes of each questionnaire (Table 1).

**Table 1:** Questionnaires and assessment tools for DED and ocular symptoms.

	Questionnaires	Description	Validation/Reliability
Subjective ocular symptoms measure	McMonnies Questionnaire (MQ) (29–33)	1. A pioneering PRO questionnaire for DED (1986). 2. Screens for possible dry eye symptoms and risk factors. 3. Evaluates the severity of eye symptoms, associated medical conditions, and treatment strategies. 4. A positive correlation exists between disease severity and the MQ.	1. Fair to moderate effectiveness. 2. Gothwal et al. indicated that the MQ is unsuitable for assessing DED severity.
	Ocular Comfort Index (OCI) (34, 35)	1. Examines patients’ recall of the severity and frequency of eye symptoms in the past week. 2. Its use as an optometric evaluation tool has been validated. 3. Can determine differences in patients’ symptoms before and after treatment.	1. The OCI is not yet validated for the subjective assessment of DED.
	Standard Patient Evaluation of Eye Dryness (SPEED) (36)	1. A 20-item questionnaire administered at three time points (now/in 72 h/in the past 3 months). 2. Evaluates the frequency and severity of symptoms on a Likert scales ranging from 0 to 3 and from 0 to 4, respectively.	1. Proven to be repeatable and valid for measuring DED symptoms and MGD-related DED.
	Symptom Assessment in Dry Eye (SANDE) (37)	1. Quantifies the severity and frequency of symptoms. 2. Uses the visual analog scale (VAS) format. 3. Two versions of assessments were designed. 4. Version 1: Initial clinical evaluation and symptom severity examination. 5. Version 2: Comparisons are performed with version 1 performed 2 months later.	1. Satisfactory repeatability when evaluation was performed. 2. The SANDE can determine changes in dry eye symptoms and can be used as a rapid and valid method to evaluate the frequency and severity of symptoms.
Quality of life assessment	Dry-Eye-Related Quality of Life Score (DEQS) (26, 38, 39)	1. A 15-question form that emphasizes the effects of DED on patients’ QoL. 2. It assesses the frequency and severity of subjective symptoms and evaluates the effects of DED on patients’ daily life.	1. Satisfactory validity and reliability 2. The DEQS is validated in the Thai and Japanese populations
	Dry Eye Questionnaire (DEQ) and DEQ-5 (32, 40–42)	1. The DEQ quantifies the severity of DED by examining the degree and frequency of symptoms. 2. Unlike other questionnaires, the DEQ has a recall period of 1 week for assessing the diurnal severity of ocular symptoms. 3. A shorter version consisting of five questions (DEQ-5) was created by modifying the DEQ.	1. The DEQ exhibited positive correlations with the MQ and OSDI, but its reliability was not proven. 2. The DEQ-5 is an effective diagnostic tool for DED.
	Contact Lens Dry Eye Questionnaire (CLDEQ) (43, 44)	1. A derivative of the DEQ designated for contact lens wearers. 2. A self-administered instrument for screening dry eye symptoms under the circumstances of wearing contact lenses. 3. A shorter version, CLDEQ-8, is available. 4. The CLDEQ-8 examines the frequency of discomfort and removing contact lens to relieve discomfort.	1. Its accuracy in discriminating between normal and contact-lens-related dry eyes was validated in comparison with the MQ. 2. The CLDEQ-8 exhibited an excellent dose–response relationship with patients’ feeling for soft contact lenses.
	Ocular Surface Disease Index (OSDI) (45–50)	1. The OSDI is the most frequently used instrument, and the committee has reached a consensus on the use of the OSDI for QoL assessment in patients with dry eye. 2. It evaluates ocular irritation symptoms caused by DED and its effect on visual function in daily life over the past week. 3. The OSDI comprises three subscales, assessing the frequency of ocular symptoms, vision-related impact on the quality of life, and environmental triggers, encompassing a total of 12 questions.	1. The OSDI exhibited satisfactory validity and reliability for measuring the severity of DED. 2. The OSDI is useful for distinguishing patients with DED from normal individuals. 3. The OSDI has been validated in different languages, although their cutoff values differ.
	Impact of Dry Eye on Everyday Life questionnaire (IDEEL) (51–54)	1. This scale assesses DED across several relevant domains: impact of QoL related to physical functioning in vision, mental perspectives, and work-related effects. 2. It has a three-module, 57-question form: discomfort caused by DED symptoms, effect of DED on daily life, and treatment satisfaction.	1. Developed and validated by Abetz et al. 2. The-disease specific IDEEL outperformed generic health questionnaires in distinguishing severity levels, with good reliability in differentiating patients with DED from normal individuals.

### DED treatments

AT and ointments are commonly used as first-line therapy 1 (55, 56). They are available in various formulations with different active ingredients, electrolyte compositions, osmolarity, and viscosities (57). These formulations may contain viscosity-enhancing agents, electrolytes, osmoprotectants, oily compounds, antioxidants, and preservatives. Oily agents and surfactants supplement the tear film lipid layer. Antioxidants, such as vitamin A and vitamin E, are integrated to address oxidative stress associated with DED (58, 59).

#### Tear supplements: active ingredients

Polymeric composites are commonly incorporated into artificial tears due to their hygroscopic and mucoadhesive properties. One advantage is the enhancement of tear viscosity, which prolongs the duration of tear retention on the ocular surface and maintains smooth tear distribution (60). Among the listed ophthalmic demulcents, carbomer, also known as polyacrylic acid, is an earlier additive used to increase the viscosity of artificial tears; its capacity to prolong ocular hydration has been reported (61). Enhancing the tear remnant improves TBUT and fluorescein test results, reduces subjective symptoms (62, 63), and improves patients’ QoL (64, 65). Since then, polymeric composites have been used to alleviate the symptoms of DED (Table 2).

**Table 2:** Tear supplement: active ingredients.

Active ingredients	Description	Comparisons
Hydroxypropyl methylcellulose (HPMC)	1. Advantages: HPMC in tear supplements can prolong moisture retention on the ocular surface. Symptoms such as eye soreness, dryness, and grittiness were improved. OSDI was decreased (57.3%) in a 4-week trial course (66–68).	
Carboxymethylcellulose (CMC)	1. CMC is an anionic cellulose polymer used for its hydrophilic property and fluid retention ability. It is also a viscosity-enhancing agent, which replenishes and maintains the mucin layer for DED caused by mucin deficiency. 2. Advantages: It can improve the ocular surface condition and stabilize the precorneal tear film. CMC-containing artificial tears reduce biomarkers associated with DED and the frequency of subjective symptoms in patients with DED (69–75).	1. Compared with other demulcents, CMC was associated with greater soothing effects, decreased stickiness, and less blurring. Additionally, CMC was the preferred choice in patients with a depleted tear volume.
Hyaluronic acid (HA)	1. HA serves as a lubricant in ophthalmic demulcents and possesses hygroscopic and biocompatible properties. 2. HA inhibits oxidative damage in cells, thus supporting wound healing and reducing inflammation. 3. Advantages: 0.1, 0.15, 0.3, and 0.4% of HA ophthalmic solution all led to significant improvements in both objective symptoms and subjective OSDI scores. 0.2% HA ophthalmic solution enhanced QoL and reduced OSDI scores after 1 month of treatment (76–80).	1. A meta-analysis of the efficacy of HA eye drops in comparison with non-HA-based eye drops revealed significant improvements in tear production and stability.
Gelling Agents: Hydroxypropyl guar (HPG)	1. Being introduced into AT to create protective and lubricative gel-like layers on the ocular surface, which stabilizes the tear film’s integrity, prevents moisture loss, and reduces osmolarity of the tear film. 2. Advantages: HPG-containing formulation can improve symptoms and QoL. Improved ocular surface protection and decreased tear film evaporation were noted when using HPG teardrops. 3. The incorporation of polyethylene glycol (PEG)/propylene glycol (PG) with HPG was reported to be effective, safe, and convenient over a decade of use. 4. New products, such as PG/HPG nanoemulsion, were developed. Several studies have demonstrated patients exhibiting good tolerance toward these products (68, 72, 81–90).	1. HPG as a demulcent reduced disease severity and decreased patients’ OSDI scores and thus outperformed CMC-containing tear drops. 2. Gelling agents were comparable with HPMC-containing artificial tears in reducing OSDI scores, and they even exhibited greater consistency in improving objective symptoms.
Xanthan gum (XG)	1. XG, which is mostly combined with chondroitin sulfate (CS), is a complex polysaccharide newly utilized as a tear film stabilizer. 2. Its chemical structure can react with reactive oxygen species, indicating its role as an antioxidative molecule. 3. Advantages: It was proven to protect the ocular surface from oxidative stress, thereby preventing inflammation and reducing DED symptoms (91–93).	1. XG outperformed HPG by significantly reducing OSDI scores for subjective symptoms. 2. XG/CS tear drops were as effective as HPG-based artificial tears.
Lipid additives	1. Lipid additives were introduced to replenish the integrity of all tear film layers. 2. Lipid-containing tear products utilize liposomal components in oil substances, such as castor, olive, and mineral oils. 3. Advantages: These lipid additives were associated with more improvement in dry eye symptoms and signs, including tear retention and better IDEEL scores, especially in MGD-related DED. They were found to be beneficial either alone or in combination with other compounds to improve dry eye symptoms. Liposomal eye drops can reduce OSDI scores in patients with both evaporative and nonevaporative dry eye (94–102).	1. Recent studies have focused on testing compound eye drops that combine various active ingredients with liposomal substances, such as castor oil or mineral oil. Both combinations yielded comparable improvements in patients’ QoL. Furthermore, artificial tears containing flaxseed oil reduced OSDI scores.

#### Osmoprotectants

The hyperosmolarity of the tear film enhances inflammatory responses, leading to the morphological damage of ocular surface cells such as apoptosis of cells of the conjunctiva and cornea. The hyperosmolarity also triggers inflammatory cascades that contribute to further cell death, including loss of mucin-producing goblet cells. These reactions exacerbate DED symptoms (112). Conventional methods for addressing hyperosmolarity in DED involve the use of hypotonic tear substitutes, which exhibit a relatively brief duration for 1-2 minutes. Recently, new formulations of artificial tears have been created, incorporating one or more osmoprotectants. Table 3 contains the types of osmoprotectants that have been utilized.

**Table 3:** Types of osmoprotectants (OsPrs).

OsPrs	Description	Other highlights
L-carnitine /erythritol/glycerin	1. Osmoprotectants maintain the osmolarity of ocular surface cells, protect them from hyperosmotic stress and thereby impede the progression of DED. Osmoprotectants also prevent the apoptosis of corneal and conjunctival epithelial cells that is caused by hyperosmolarity. 2. OsPrs are often combined with CMC. 3. Advantages: When used in combination with 0.5% CMC, OsPrs reduces dry eye symptoms, improves OSDI scores, and enhances comfort and ease of use among patients. When used in combination with 1% CMC, OsPrs significantly improves OSDI in severe DED.	1. OsPr groups (erythritol/glycerin-containing formulations) exhibited more improvements than did the CMC group with a rapid and consistent reduction in subjective symptoms. 2. The OsPr demulcent was considered effective in alleviating subjective symptoms and preventing postoperative dry eye discomfort in patients with postrefractive surgery DED (81, 103–107).
Trehalose	1. Trehalose is a disaccharide with anti-inflammatory and osmoprotective properties; it inhibits the inflammatory cascade and stabilizes ocular surface cells against hyperosmotic stress. 2. Advantages Trehalose + flaxseed oil in ATs markedly reduces OSDI scores with few adverse events. Trehalose +0.1% sodium hyaluronate (SH) leads to greater improvements in Schirmer’s test results and TBUT than did SH alone. ATs containing trehalose and HA reduce OSDI and subjective symptoms.	1. Small-molecule OsPrs can enter cells to balance osmotic stress, whereas the large-molecule OsPrs act likely at the level of the cell membrane. Both small-molecules (L-carnitine, erythritol) and large-molecules (trehalose) OsPrs can elicit direct anti-inflammatory/antioxidative effects following hyperosmotic stress and have a direct benefit on DED (108–111).

#### Topical secretagogues

##### Topical immunomodulators

Topical immunomodulators have been used because of their ability to disrupt the inflammation pathway (Table 4) (140). Although topical corticosteroids can effectively disrupt the inflammatory and immune response cycle of DED, their long-term use can cause complications, such as ocular hypertension and opportunistic infections (141, 142). Tetracyclines are broad-spectrum antibiotics that possess anti-inflammatory properties. They are occasionally prescribed to treat disorders associated with DED. However, the long-term risks and safety of their use are still not well understood (141). Table 5 lists the effective topical immunomodulators, which had been applied clinically.

**Table 4:** Topical secretagogues.

Topical secretagogues	Description	Other highlights
Diquafosol	1.Diquafosol sodium exhibits a P2Y2 agonist activity that it stimulates mucin secretion from goblet cells and fluid secretion from conjunctival epithelial cells, thereby increasing tear content and hydrating the ocular surface. 2.Advantages: The clinical efficacy of 3% diquafosol ophthalmic solution has been confirmed for dry eye, including aqueous-deficient dry eye, short TBUT-type dry eye, and post-LASIK dry eye. Better TBUT and DEQS scores; significant alleviation of DED symptoms. Relief from ocular fatigue, dryness, discomfort, and foreign body sensation in patients with aqueous-tear deficiency and post-cataract surgery dry eye. It ameliorates the signs and symptoms of dry eye with SS in comparison with application of SH and AT. It reduces DEQS scores in soft contact lens-induced dry eye. 3.Disadvantages Compared with AT, diquafosol results in increased ocular adverse events (113–128).	1. No evident superiority for 3% diquafosol ophthalmic solution over 1% HA artificial tears(AT). 2. Compared with AT group, diquafosol group experienced more substantial relief from foreign body sensation. 3. The combination of diquafosol and AT did not provide notable benefits over diquafosol monotherapy, but the dual treatment might help reduce adverse events compared with diquafosol alone.
Rebamipide	1.Rebamipide is a mucoprotective agent originally used as a gastric protectant for gastric and duodenal ulcers. It effectiveness is attributed to its ability to increase mucin and thus stabilize the tear film. 2.Advantages: 1 and 2% rebamipide: Improves objective and subjective symptoms, such as foreign body sensation, dryness, photophobia (only in 2% rebamipide), eye pain, and blurred vision. 2% rebamipide: Better outcomes were observed for symptoms, including grittiness, pain, and soreness, and daily scenarios, such as reading, low-humidity environments, and air-conditioned spaces. Objective improvements (2% rebamipide): Better DEQS scores, TBUT, and fluorescein staining scores. Rebamipide was proven to have a well-tolerated safety profile (129–137).	1. Compared with 0.1% HA, 2% rebamipide shows more substantial improvements in subjective symptoms and better treatment outcomes. 2. Rebamipide can also be used in contact lens–related dry eye, where improvements were observed in all 12 OSDI items.
More recent studies of topical secretagogues	1. Both diquafosol and rebamipide were effective in enhancing patients’ overall QoL (evaluated by using DEQS). 2. New topical secretagogues are still developing, such as eledoisin, 3-isobutyl-1-methylxanthine, recombinant human nerve growth factor, and MIM-D3 (a small-molecule nerve growth factor peptidomimetic). 3. For rhNGF, a phase IIa, open-label, multiple-dose study indicated that both doses of 20 and 4 μg/mL are safe and effective in improving the symptoms and signs of DED (126, 138, 139).	

**Table 5:** Topical immunomodulators.

Topical immunomodulators	Description	Comparisons and adverse events
Cyclosporine A (CsA)	1. CsA reduces the severity of dry eye by inhibiting T-cell proliferation and downregulating inflammatory pathway signals. 2. Advantages: Various doses of CsA ophthalmic solution significantly improved OSDI scores. Improves subjective symptoms and reduces the dependence on artificial tears (AT). Objective parameters: Better corneal and conjunctival staining scores, OSDI scores, Schirmer values, and TBUT. Sjögren syndrome: 0.05% topical CsA improved subjective symptoms. Contact lens wearers with DED: 0.05% CsA ophthalmic emulsion improved subjective symptoms and OSDI scores (143–150).	1. The effectiveness of CsA may decrease when used in combination with ATs. 2. Compared to AT, CsA exhibits better TBUT, fluorescein-staining scores, and OSDI scores. 3. CsA resulted in more adverse events than ATs, even though none of them were severe. 4. Restasis takes up to 3 months to begin reducing dryness.
Lifitegrast	1. Lifitegrast, a lymphocyte function–associated antigen 1 (LFA-1) antagonist, alleviates inflammation by inhibiting T-cell recruitment, T-cell activation, and subsequent cytokine release. 2. Lifitegrast improved DED signs in patients with mild-to-moderate disease (phase 2 and OPUS-1 studies) 3. Lifitegrast improved DED symptoms in moderate-to-severe disease (OPUS-2 study). 4. *Post hoc* analysis of OPUS-2 and OPUS-3 trials demonstrated a twofold-higher odds of achieving significant improvement in moderate-to-severe DED patients.	1. LFA-1: the incidence of adverse events slightly differed from that in the placebo, especially instillation site discomforts and dysgeusia. 2. Lifitegrast can begin reducing eye dryness within 2 weeks, whereas Restasis takes up to 3 months. 3. The users of CsA and lifitegrast reported ineffective relief of DED symptoms (31 and 22%, respectively) and dissatisfaction with the time to onset of effect (29 and 11%). 4. In both groups, one- third of patients experienced unsuccessful relief from symptoms (151–157).
Reproxalap	1. A novel reactive aldehyde species inhibitor that binds to free aldehyde targets. 2. Well tolerated and effective in mitigating the symptoms and signs of DED (158).	1. Current studies still lack more reliable evidence, necessitating further research to confirm its efficacy and safety.

##### Biological tear substitutes

Blood-derived topical products were first used to treat ocular surface disease by Ralph et al. in 1979 (159). Since then, serum eye tears have been used to treat DED in clinical practice (Table 6).

**Table 6:** Biological tear substitutes.

	Description	Comparisons and adverse events
Autologous serum (AS)	1. It is the most utilized biological tear substitute, and its composition resembles that of human tears. It also consists of rich beneficial ingredients, such as vitamin A and C, lysozyme, growth factors, and fibronectin. This helps AS replenish the tear-impaired ocular surface resulting from DED. 2. Advantages: It improves the QoL of patients with DED by improving OSDI and SANDE scores and alleviating subjective symptoms (lower VAS scores). For patients with SS, improvements can be observed in subjective symptoms, such as burning, foreign body sensation, and dryness. All AS formulations reduced subjective symptoms. Furthermore, 100% AS was reported to have a better response over diluents of 50% AS. 3. Overall, AS treatment led to high treatment satisfaction and convenience. 4. Finger-prick AS has better availability, which led to improvements in OSDI scores, ocular surface staining scores, and Schirmer test results compared with conventional treatment in patients with moderate to severe dry eye. 5. Currently, available studies have reported the short-term benefits of AS for enhancing the QoL of patients with DED but have failed to prove efficacy in longer periods (160–175).	1. A combination of 0.05% CsA and ATs is superior to 20% AS in improving Schirmer test results and TBUT scores in patients with SS. 2. Limitations in these hemoderivative treatments include the cost or availability. 3. Nonsignificant objective parameters of TBUT were noted in one of the studies. 4. Few complications have been reported, such as eye discomfort, epitheliopathy, microbial infections, and eyelid eczema.
Other types of biological tear substitutes	1. For people who are unable to donate their own blood for AS, allogeneic serum (HS) and umbilical cord sera (CS) may be alternative options. 2. All three treatments (AS, HS, and CS) demonstrated significant effects on visual acuities, Schirmer test results, TBUT, fluorescein and lissamine green staining measurements and questionnaire scores. 3. More studies are still being conducted to find more evidence on these biological substitutes, and further trials are needed to define their efficacy and safety (126, 176, 177).	1. AS led to more favorable improvements in OSDI scores than HS. 2. Biological substitutes^1^ might be the most effective treatment among tear-promoting eye drops^2^ in relieving dry eye symptoms without increasing adverse effects^3^.

^1^AS, cord blood serum, autologous platelet lysate, platelet-rich plasma. ^2^Including biological substitutes and topical secretagogues. ^3^These findings were obtained from primary studies with low quality and sparse data, which led to evidence uncertainty.

##### Nutritional intervention

Previous studies have explored the use of nutritional strategies to improve DED. A novel botanical combination of lutein ester; zeaxanthin; and extracts from blackcurrant, chrysanthemum, and goji berry was designed to treat adults with eye fatigue. This formula ameliorated eye soreness, blurred vision, dry eye, foreign body sensation, and increased tearing, resulting in enhanced scores on questionnaires used to evaluate dry eye conditions (178).

#### Procedures

Punctal occlusion can reduce the drainage of tears into the lacrimal ducts, thereby conserving tears, providing lubrication, and alleviating dry eye symptoms (179). Many types of plugs, including those made of silicone and collagen, have been investigated. Improvements in irritative symptoms, as well as reductions in central, superior, nasal, and temporal corneal staining were noted DED patients with bilateral punctal plug insertion (Table 7).

**Table 7:** Procedure options for DED.

Types of procedures		Description	Other Highlights
Punctal occlusion	Silicone and Collagen plugs	1. Collagen plugs, which dissolve in 4–7 days, were typically initiated. If collagen plugs were reported to be effective, plugs of more permanent materials, such as silicone or acrylics, would then be inserted. 2. Advantages: Collagen and silicone punctal plugs both benefited patients by relieving seven individual symptom scores^1^ Patients with post-LASIK dry eye: Numerous types of punctal occlusion all showed effectiveness in reducing OSDI scores, symptoms of dryness and foreign body sensation to enhance patients’ QoL. 3. Limitations: Fewer patients exhibited subjective improvements in symptoms, such as decreased OSDI scores, in silicone plug (37.5%) compared with smart plugs (thermosensitive, 95.5%). Spontaneous plug loss is among the largest obstacles for silicone plugs (180–185).	1. No significant differences in subjective questionnaire and objective measurements (tear film thickness) were observed between punctal occlusion and sham procedures in patients wearing contact lens.
	New types of plugs: Hydroxybutyl chitosan (HBC)	1. It is a new “liquid plug” strategy involving intracanalicular injection of HBC solution, which is a thermosensitive and phase-changing biomaterial. 2. Advantages: HBC relieved the symptoms and signs of DED. Improvements were noted in OSDI scores, phenol red test results, and tear meniscus height. 3. Overall, HBC injection showed promising efficacy and safety and thus may be an alternative for punctal occlusion (186).	
	Adverse events and evidence	1. Foreign body sensation, epiphora, spontaneous plug loss or displacement, and itchiness at plug placement sites have been reported. 2. However, a systematic review indicated that although punctal plugs are effective means for treating dry eye signs and symptoms, evidence regarding improvements in symptoms and commonly tested dry eye signs remains inconclusive (187–189).	
Botulinum toxin type-A (BTX-A)	Injection of BTX-A to medial orbicularis muscle of lower eyelid	1. Advantages: In the group with lower eyelid injection, the median lacrimal drainage capacity after 3 weeks was reduced to 52% of baseline level. In the group with upper and lower eyelid injections to 42%. An RCT: injection of BTX-A in the medial orbicularis muscle portion of the lower eyelid can improve symptoms and signs of DED. An RCT of injection of BTX-A or normal saline in the medial part of the upper and lower eyelids reveals the MMP-9 conversion rate was significantly higher and the tear serotonin level was significantly reduced in the BTX-A injection group than that of the normal-saline injection group (190–193).	1. The improvement following BTX-A injection disappeared within 3 months. 2. Two out of 10 subjects reported epiphora which occurred in situations with reflex lacrimation due to BTX-A injection in both the upper and lower eyelids which disappeared after 1 month.
	Injection of BTX-A to Horner’s Muscle	1. Advantage: In 2 cases, a significant improvement was observed in the subjective perception of the patient, the OSDI, superficial punctate keratitis, and the time of the tear rupture and tear meniscus at 1 month after treatment, with an acceptable response still being maintained at the third month (190, 194).	
Thermal pulsation	Vectored thermal pulsation (VTP)	1. Advantages: Sjögren’s syndrome-related DED: VTP can improve the meibomian gland oil flow scores, corneal and conjunctival staining scores, and TBUT. Patients with MGD: A single session of VTP was effective in improving objective signs^2^. These benefits are associated with better OSDI, SPEED, and SANDE scores. 2. Limitations: As the observation period becomes longer, TBUT and OSDI returned to baseline level. 3. In a Chinese study examining the effect of 12-min VTP, the SPEED score and TBUT improved from baseline with better lipid layer thickness and meibomian gland secretion scores (195–203).	
	Comparisons and applications of VTPs	1. VTP versus warm compress Efficacy and safety: Comparable outcomes were noted for single-dose VTP and 3 months of twice daily warm compress in Asian patients. 2. VTP versus oral doxycycline Both improved MG function, TBUT, corneal and conjunctival staining scores The VTP group exhibited better SPEED scores. 3. For patients with recalcitrant dry eye who underwent LASIK and photorefractive keratectomy (PRK), SPEED scores were improved when they received single-dose VTP (195, 196, 201, 204, 205).	
	VTP systems	1. Because most researchers have used the LipiFlow system to evaluate the effectiveness of VTP, Systane iLux thermal pulsation treatment is a new system used to treat patients with MGD. 2. Systane iLux thermal pulsation treatment Can increase meibomian gland secretion and tear film stability and reduce dry eye symptoms. 3. LipiFlow versus Systane iLux. Comparable improvements in meibomian gland scores, TBUT, and IDEEL-SB scores were noted in patients with dry eye–associated meibomian gland dysfunction. 4. However, the aforementioned benefits of thermal pulsation procedures were not prominent in a study conducted in 2020 (206–210).	
Intense pulsed lighting (IPL)		1. Advantages: IPL can improve the lipid layer grade, TBUT, tear film osmolarity, OSDI, and visual analog scale symptom scores. Contact lens-related dry eye: Improvements were observed in the OSDI score, tear quality, and meibomian gland quality. 2. IPL and forced meibomian gland expression (MGX) When IPL was combined with MGX, tear quality and meibomian gland’s function improved within 6 months. MGX may be essential after IPL. Patients treated with IPL may only experience a shorter time to MGD recurrence than those treated with MGX. SS–related DED: IPL-MGX considerably improved the OSDI score, NITBUT^3^, CFS^4^, eyelid margin abnormalities, MGX, and meibum quality. 3. Adverse events Mild pain and burning were reported in some patients. 4. New-generation IPL(Eyesis) It has a noninferior effective rate than traditional IPL (E-Eye). It demonstrated more clinical benefits over E-Eye in relieving symptoms, increasing tear film stability, and improving meibomian gland function (211–226).	
Minor Salivary Gland Transplantation (MSGT) and Submandibular Gland Transplantation (SMGT)		1. MSGT The volume of the resulting lubrication is very limited in severe DED. Long-term improvement in the visual acuity, ocular surface environment, and keratopathy can be found. Reflex epiphora is rarely a problem in MSGT. 2. SMGT SMG produces a more tear-like, seromucous secretion. SMGT is a lasting and effective solution for patients with severe DED. Provide abundant lubrication in severe DED. Possible complications: blood vessel thrombosis, Wharton’s duct obstruction, and epiphora are surgical complications (227–238).	

^1^Dryness, watery eyes, itching, burning, foreign body, fluctuating vision, and light sensitivity. ^2^Tear osmolarity, TBUT, corneal staining score, and meibomian gland evaluation scores. ^3^Noninvasive tear break up time. ^4^Corneal fluorescein staining.

Botulinum toxin type A injection in the medial part of the lower eyelid is considered an alternative method of punctal occlusion to reduce lacrimal drainage (239). Botulin toxin type-A (BTX-A) can demonstrate less lacrimal clearance by denervating lacrimal part of orbicularis oculi muscle. This procedure can be done by injecting BTX-A into upper or lower eyelids. Injection in the lower eyelid alone showed better improvements than injection in both the upper and lower eyelids. However, the effect cannot last long in most patient with a range of 3 months (240).

Vector thermal pulsation (VTP) can provide warm compress to the eyelids and meibomian gland (241). Thermal pulsation has many advantages, with potentially the longest-lasting per-treatment effect for MGD (206). Intense pulsed lighting involves the application of highly intensified pulses of polychromatic light across a broad wavelength range (515–1,200 nm) for eliminating superficial capillary vessels in the periocular region, reducing the release of tear inflammatory cytokines, and improving the outflow of the meibomian gland (211). However, because of the paucity of high-quality research, the effectiveness and safety of long-term intense pulsed lighting treatment for MGD remain uncertain, necessitating further research (242).

Salivary gland transplantation should be considered to treat severe DED. Submandibular gland transplantation (SMGT) and minor salivary gland transplantation (MSGT) are the most commonly used procedure, while parotid gland was proved to be non-beneficial for severe DED (227, 228). Previous studies demonstrated autologous microvascular SMGT improved objective signs and subjective symptoms of severe DED (229, 230, 243). Su et al. conducted a prospective study and revealed that the significant improvement of life quality and satisfaction of DED patients after SMGT (231). Although SMGT and MSGT provided benefits for severe DED patients, SMGT should be recommended to treat end-stage refractory DED (232). Table 8 compiles a summary of various treatment options for DED, encompassing tear supplements, osmoprotectants, secretagogues, immunomodulators, biological tear substitutes, and procedures.

**Table 8:** Treatment options for DED.

Active ingredients	Hydroxypropyl methylcellulose (HPMC)	
	Carboxymethylcellulose (CMC)	
	Hyaluronic acid (HA)	
	Hydroxypropyl guar (HPG)	
	Xanthan gum (XG)	
	Lipid additives	
Osmoprotectants (OsPrs)	L-carnitine/erythritol/glycerin	
	Trehalose	
Topical secretagogues	Diquafosol	
	Rebamipide	
	Novel therapies	Eledoisin
		3-Isobutyl-1-methylxanthine
		Recombinant human nerve growth factor
		MIM-D3
Topical immunomodulators	Cyclosporine A (CsA)	
	Lifitegrast	
	Reproxalap	
Biological tear substitutes	Autologous serum (AS)	
	Other types of biological tear substitutes	Allogeneic serum (HS)
		Umbilical cord sera (CS)
Procedure options	Punctal occlusion	Silicone and Collagen plugs
		Hydroxybutyl chitosan (HBC)
	Botulinum toxin type-A (BTX-A)	Injection to medial orbicularis muscle of lower eyelid
		Injection to Horner’s muscle
	Thermal pulsation	Vectored thermal pulsation (VTP)
	Intense pulsed lighting (IPL)	
	Salivary gland transplantation	Submandibular gland(SMGT)
		Minor salivary gland (MSGT)
Nutritional intervention	Botanical combination of lutein ester/zeaxanthin/extracts from blackcurrant, chrysanthemum, and goji berry.	

## Conclusion

In our investigation of various treatments and questionnaires for DED, we found multiple validated questionnaires designed to collect PROs. Although inconsistencies exist between questionnaires and clinical findings, they provide valuable information in the initial evaluation and monitoring of DED treatments. However, the lack of standardized measures and intergroup conversion causes difficulty in cross-comparison.

The initial therapy of DED involves the use of artificial tears. Over-the-counter formulations containing ingredients, such as CMC and HA, and osmoprotectants, such as trehalose, aim to restore hydration and lubrication, thereby alleviating dry eye symptoms (69, 244). However, in advanced cases of moderate-to-severe dry eyes, artificial tears might not be effective (245). Liposomal tear drops are beneficial for both evaporative and non-evaporative DED (94), and secretagogue drops can improve the QoL of patients with short TBUT or aqueous deficient-type DED (113).

Immunomodulators provide rapid symptom relief, and most patients with DED report the effectiveness and high satisfaction rate of cyclosporine A (CsA) and lifitegrast (246–248). However, trials have consistently reported adverse events, such as irritation or pain at the instillation site, which may affect patient compliance and therapy efficacy (249, 250).

Patients who received autologous serum (AS) treatments reported high satisfaction and expressed eagerness to continue the therapy (251). However, well-established production and storage protocols are still needed for their clinical use (160, 161). Punctual occlusion has been performed to either temporarily or permanently block tear drainage from the lacrimal punctum. However, this procedure is associated with a higher complication rate (even up to 60%) (252), making it a less favorable option for treating DED (239). BTX-A serving as a temporary solution for DED, it can significantly improve symptoms within 3 months by reducing lacrimal drainage (240).

VTP is a novel therapy option, particularly for DED caused by MGD (195, 253). VTP offers a convenient solution for individuals with MGD-related dry eye, representing an alternative treatment option for patients with modern busy lifestyles (254). Intense pulsed light(IPL) also has proven to be effective for treating evaporative dry eye caused by MGD (255), with 93% of patients reporting posttreatment satisfaction without any severe adverse effects. Multiple studies have confirmed the efficacy of combining IPL treatment with meibomian gland manipulation (256).

In severe DED cases, SMGT offers a promising approach for tear film restoration (243). Previous studies demonstrated autologous SMGT has a high success rate, and it significantly improved quality of life and satisfaction (231).

Overall, patient satisfaction and QoL evaluations often improved after different DED treatment modalities. This review highlights the importance of PRO assessments for evaluating the effect of DED treatments on subjective symptoms and QoL. Given that the assessment of DED treatment effectiveness substantially relies on individual ocular experiences, acquiring qualitative PRO data is essential for comprehensive evaluation and optimal treatment management. Clinicians should not only focus on improving objective symptoms but also prioritize the well-being of patients in clinical settings.

## Author contributions

C-WL: Investigation, Writing – original draft. M-YL: Conceptualization, Formal analysis, Supervision, Visualization, Writing – review & editing. J-WH: Conceptualization, Formal analysis, Methodology, Resources, Writing – review & editing. T-JW: Writing – original draft, Investigation, Methodology, Project administration, Visualization. I-CL: Methodology, Supervision, Writing – review & editing.
